# Online survey of university students’ perception, awareness and adherence to COVID-19 prevention measures

**DOI:** 10.1186/s12889-022-13356-w

**Published:** 2022-05-13

**Authors:** Salma Akhter, Meredith Robbins, Perry Curtis, Belle Hinshaw, Ellen M. Wells

**Affiliations:** 1 School of Health Sciences, Purdue University, West Lafayette, IN USA; 2 Department of Public Health, Purdue University, West Lafayette, IN USA

**Keywords:** Policy adherence, Prevention, Students, COVID-19, Masks, Social distancing

## Abstract

**Background:**

Determining factors correlated with protective measures against COVID-19 is important to improve public health response. This study describes student opinions related to university COVID-19 preventive measures.

**Methods:**

In fall 2020, 643 US university students completed an online survey on perception, awareness, and adherence to COVID-19 preventive measures. Outcomes included protocol effectiveness (self or others), protocol adherence (self or others), consequences of protocol violation, knowledge of violations, and level of concern for COVID-19. Multiple linear regression models determined correlates of outcome variables. Covariates included gender, race, residence, area of study, class, and knowledge of someone with a positive COVID-19 test.

**Results:**

Overall, students agreed with protective measures (equivalent to higher scores). In adjusted linear models, females (versus males) had significantly higher scores for protocol effectiveness (self) (*p* < 0.001), consequences of protocol violation (*p* = 0.005), and concern about COVID-19 (*p* < 0.001). Asian/Pacific Islander (versus white) had significantly higher scores for protocol effectiveness (self) (*p* < 0.001), consequences of protocol violation (*p* = 0.008), and concern about COVID-19 (*p* = 0.001). Graduate students (versus freshman) had higher scores for protocol effectiveness (self) (*p* < 0.001), protocol adherence (self) (p = 0.004) and concern about COVID-19 (*p* < 0.001). In contrast, participants who had a positive COVID-19 test had significantly lower scores for protocol effectiveness (self) (*p* = 0.02), protocol adherence (self) (*p* = 0.004), and consequences of protocol violation (*p* = 0.008).

**Conclusion:**

Overall, females, Asian/Pacific Islanders, and graduate students were more likely to agree with or adhere to COVID-19 prevention guidelines but those who tested positive for COVID-19 were less likely to do so. These results may inform future prevention efforts.

**Supplementary Information:**

The online version contains supplementary material available at 10.1186/s12889-022-13356-w.

## Background

COVID-19, a respiratory infection caused by SARS-CoV-2, was first described in late 2019 and identified as an international public health emergency in January 2020 [1–3]. Response to COVID-19 included rapid development of protocols for diagnosis, treatment, containment and prevention efforts [2]. A variety of different prevention measures were implemented, including closures, virtual events or telework, social distancing, mask wearing, and surveillance programs [2, 4–7].

However, effective implementation of the majority of these measures relies on individual behavior change, which can be challenging. Prior to the pandemic, reported rates of compliance with standard infection control prevention policies among hospital staff were reported between 50–70% [8–10], and even less than 50% [11]. A recent review of 56 papers found staff compliance with preventive measures was associated with working in emergency or ICU settings as well as high level of risk perception and concern [12]. This raises concern that compliance among individuals in other settings, where adherence would not affect employment, could be even lower.

In order to facilitate better adherence, it is important to understand characteristics correlated with adherence. In studies prior to the COVID-19 pandemic, females were more likely to wear masks in the early stage of the H1N1 influenza epidemic [13] and had a higher perception of risk compared to males [14]. Those with more education were more likely to wear masks in public areas [13]. Non-white participants, compared to white study participants, were more likely to follow protective guidelines [15] or indicate a willingness to get an influenza vaccine [16]. A systematic review of 26 studies during the swine flu pandemic showed that female, older, more educated and non-white participants adopted protective behaviors more than other demographic groups [17].

These findings are largely consistent with studies conducted during the COVID-19 pandemic. Females tended to follow the suggested guidelines (e.g., social distance, hand hygiene practice) more than males, and their perceived risks from COVID-19 were also higher than that of their male counterparts [18–21]. Additionally, respondents from countries that were severely affected by COVID perceived protective measures as more important than that from countries that were less affected [21, 22].

At the time this research was initiated, there were very few reports of attitudes towards prevention among students, which is a notable gap as student perceptions and adherence to prevention guidelines may vary substantial from other groups. However, since the start of the COVID-19 pandemic, research has explored the rate of students’ compliance with preventive measures [23–26] as well as attitudes or perceptions towards preventive measures among Iranian high school students [27] or among university students from the Middle East [28, 29], Asia [30, 31], Europe [32, 33], and black college students from North Carolina, USA [34]. Therefore, the purpose of this study is to add to this growing body of literature to explore the perceptions and awareness of COVID-19 prevention measures among students at a large United States university.

## Methods

### Study design

This was a cross-sectional, online survey of 643 undergraduate and graduate students at Purdue University, Indiana, USA from October 2020 through December 2020. During this time, Purdue University was among the minority of US universities to offer at least some classes in a face-to-face format and a majority of students were physically on campus.

Purdue University developed COVID-19 prevention strategies (“Protect Purdue”); under these guidelines the university operated with both online and hybrid in-person/online instruction during 2020–2021. Briefly, social distancing was required whenever feasible. Cloth mask use was required indoors and when social distancing was not possible outdoors. When social distancing was not possible indoors, face shields were required with masks. These guidelines were applied to both on- and off-campus events. All students were required to have a COVID-19 test prior to arrival on campus and to participate in random surveillance testing throughout the school year. All students, faculty, and staff signed a pledge indicating their willingness to follow these guidelines. Violators could potentially face strict penalties, including suspension [35]. The protocols were consistent throughout the data collection period.

### Study population

Eligibility criteria included 1) undergraduate or graduate student status at Purdue University; 2) ≥ 18 years old; 3) completion of informed consent. Individuals were excluded if they did not complete the full survey. Participants provided informed consent prior to completing the survey. This study was determined to be exempt from Institutional Review Board review by the Purdue University Biomedical Institutional Review Board.

Participants were recruited through flyers and direct emails. Flyers posted across campus included a URL and QR code that students could use to access the survey: *N* = 342 individuals used the URL and *N* = 121 individuals used the QR code. Direct emails were sent to a random sample of 4000 undergraduate and graduate students (8.7% of enrolled students). Of these, 473/4000 (11.8%) accessed the survey. Altogether, 936 students started the survey (Supplemental Figure S1). Participants were excluded due to not meeting inclusion criteria (*N* = 119) or not completing the survey (*N* = 171). An additional 3 participants who indicated nonbinary gender were excluded due to a lack of power to interpret this category. A total of 643 participants (69.0% of those who accessed the survey) were included in analyses.

### Study variables

The 21-item online survey covered demographics, student status, experience and perceptions of COVID-19 as well as COVID-19 prevention measures. The survey was developed by the investigators. Demographic/student status variables included gender, race/ethnicity, residency (in-state, out-of-state, or international), class, and college. Seven options were provided for race/ethnicity; however, due to low numbers in some categories this was recoded to white, Asian/Pacific Islander, or other/multiracial for analysis. Awareness/experience variables included “do you know anyone who has tested positive for COVID-19?” and “how concerned are you about COVID-19?”. Participants were also asked whether they agree or disagree with the statement “I am aware of students who are hosting social events that violate the Protect Purdue guidelines”.

Participants were asked to indicate the extent to which they agreed or disagreed with 12 statements related to COVID-19 prevention measures using a 5-point Likert scale (Supplemental Table S1). The Cronbach’s alpha for these 12 items was 0.74. Responses were scored from 1 = “strongly disagree” to 5 = “strongly agree”. Most statements were originally written so that a response of “strongly agree” was consistent with increased prevention measures or belief in the effectiveness of prevention measures. Three statements which did not meet this pattern were recoded for analyses. These 12 items were grouped into five broader categories: participant’s own perception of the effectiveness of prevention measures (“protocol effectiveness (self)”); how others perceive the effectiveness of prevention measures (“protocol effectiveness (others)”; participant’s own adherence to preventive measures (“protocol adherence (self)”); others adherence to preventive measures (“protocol adherence (others)”; and perception of protocol violation consequences (“consequences of protocol violation”).

## Data analysis

Analyses were completed using Stata Version 16 (College Station, TX); a p-value < 0.05 was considered to be statistically significant. Descriptive analyses were completed to assess the frequency and distribution of all variables. Linear (unadjusted) regression and multiple (adjusted) linear regression were used to compare predictor variables with perceptions of COVID-19 measures, knowledge of anyone holding social events which violated these measures, and self-reported level of concern about COVID-19. Predictor variables were all coded as categorical variables and included gender, race, residency, college, class and knowledge of someone with COVID-19. Multiple linear regression models included all predictor variables as covariates. Results are presented as change in perception score on a scale of 1 to 5 with a higher score indicating a higher use or belief in preventive measures.

## Results

Overall, 643 students were included in analyses (Table 1). Approximately 60% of participants were female and 62% had in-state residency. The majority of participants were white (*N* = 465, 72.3%), the remainder were Asian or Pacific Islander (*N* = 101, 16.7%) or other/multiracial (*N* = 77, 12.0%). The College of Engineering (*N* = 146, 22.7%) and College of Health and Human Sciences (*N* = 111, 17.3%) had the most participants. Most of respondents were undergraduate students (*N* = 455, 70.8%), the remainder were graduate students (*N* = 188, 29.2%). The majority of participants reported knowing someone who was COVID-19 positive (*N* = 511, 80.3%). Most students reported being “moderately concerned” about COVID-19 (*N *= 240, 37.3%), with the remainder roughly equally divided between being less concerned and more concerned. Just under half of the participants reported that they “somewhat agree” or “strongly agree” with the statement that they are aware of social events in violation of prevention policies (*N* = 305, 48.0%).

**Table 1:** Population characteristics

Category	Variable	*N*	Percent
Gender	Male	257	40.0
	Female	386	60.0
Race	White	465	72.3
	Asian or Pacific Islander	101	15.7
	Other or multiracial	77	12.0
Residency	In state	397	61.7
	Out of state	188	29.2
	International	58	9.0
College	Engineering	146	22.7
	Liberal Arts	56	8.7
	Health and Human Sciences	111	17.3
	Polytechnic Institute	55	8.6
	Science	69	10.7
	Other/more than one college	206	32.0
Class	Freshman	127	19.8
	Sophomore	125	19.4
	Junior	102	15.9
	Senior	101	15.7
	Graduate student	188	29.2
Know someone with COVID-19	No	132	20.5
	Family	37	5.8
	Friend	150	23.3
	Self	9	1.4
	Someone else	114	17.7
	Others/multiple	201	31.3
Aware of social events in violation of policy	Strongly disagree	109	17.0
	Somewhat disagree	108	16.8
	Neither agree nor disagree	121	18.8
	Somewhat agree	162	25.2
	Strongly agree	143	22.2
Level of concern about COVID-19	Not at all concerned	62	9.6
	Somewhat concerned	145	22.6
	Moderately concerned	240	37.3
	Very concerned	141	21.9
	Extremely concerned	55	8.6

**Fig. 1:**
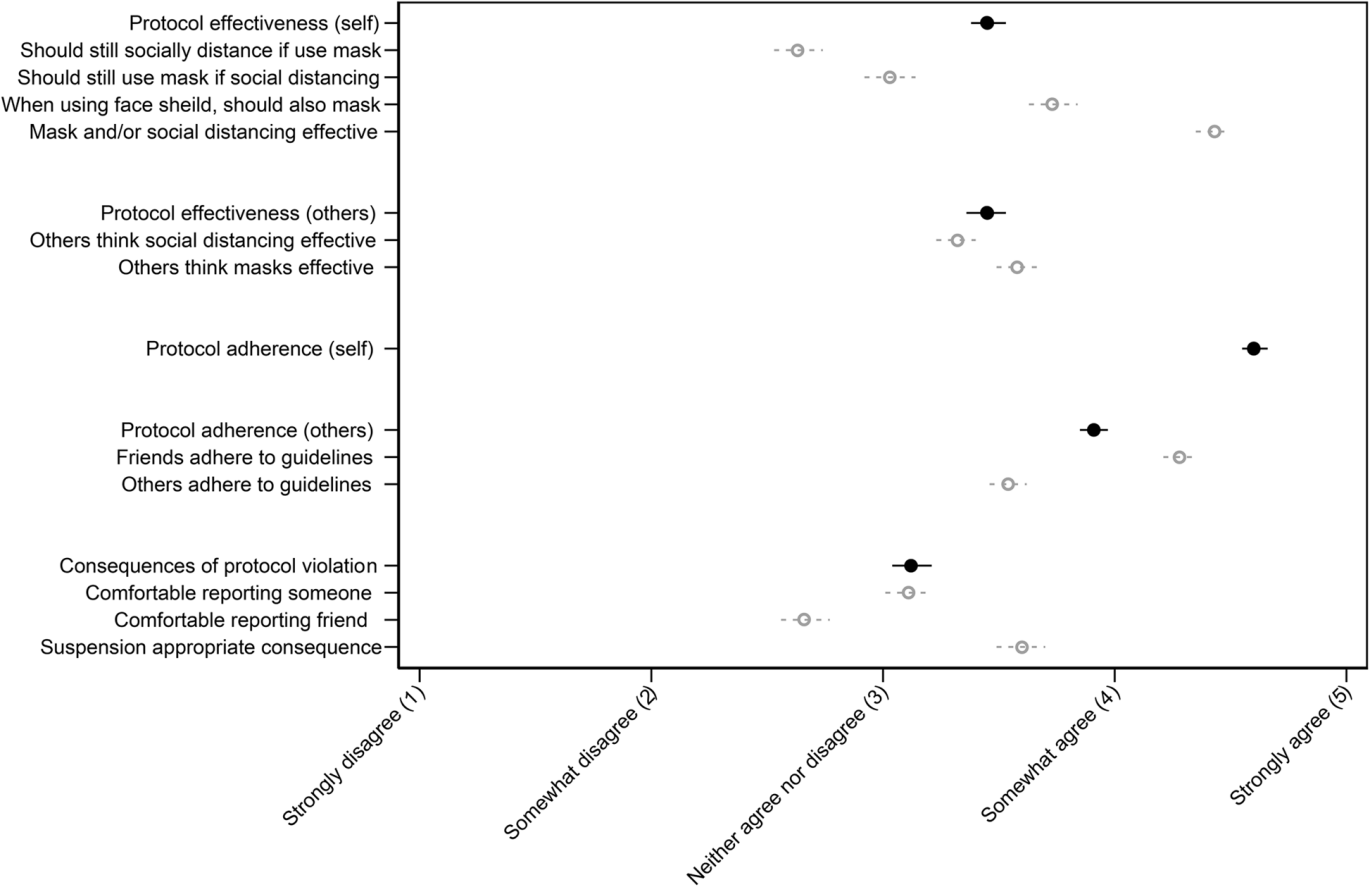
Mean scores (dot) and 95% confidence interval (line) for student’s agreement with statements about COVID-19 prevention protocols. Outlined/dashed markers indicate individual questions; black markers indicate scores for the overall category

Mean scores for the questions related to adherence or perception of COVID-19 prevention measures are presented in Table S1 and Fig. 1. “Protocol adherence (self)” (mean score: 4.60; SD: 0.71) and protocol adherence (other) (mean score: 3.91; SD: 0.78) received the first and second highest scores, respectively. Protocol effectiveness for both self (mean score: 3.45; SD: 1.01) and others (mean score: 3.45; SD: 1.09) received the next highest mean scores, and consequences of protocol violation received the lowest scores (mean score: 3.12; SD: 1.11).

Results from linear (unadjusted) regression models are presented in Supplemental Tables S2 and S3 and were similar to results from multiple linear regression (adjusted) models. All multiple linear regression models included gender, race, residency, college, class and knowledge of someone with COVID-19 as covariates; results are presented in Tables 2–3. Interactions between predictor variables were not evaluated in these models. Scores for protocol effectiveness (self) were significantly higher among women (versus men), Asian/Pacific Islander or other race/multiracial (versus white), out of state (versus in-state), as well as juniors, senior or graduate students (versus freshman) (Table 2). Those who reported having a positive COVID-19 test themselves (versus not knowing anyone who tested positive) had significantly lower scores on protocol effectiveness (self). There were significantly lower scores for protocol effectiveness (others) among the participants who knew friends, “someone else”, or multiple people who tested positive for COVID-19 (versus those who reported none).

**Table 2:** Adjusted difference in opinions on effectiveness or adherence to prevention protocols associated with selected characteristics

Category	Variable	Protocol effectiveness (self)	Protocol effectiveness (others)	Protocol adherence (self)	Protocol adherence (others)	Consequences of protocol violation
Gender	Male	*Referent*	*Referent*	*Referent*	*Referent*	*Referent*
	Female	0.32 (0.16, 0.48)***	-0.22 (-0.40, -0.03)*	0.10 (-0.02, 0.23)	-0.07 (-0.21, 0.06)	0.27 (0.08, 0.46)**
Race	White	*Referent*	*Referent*	*Referent*	*Referent*	*Referent*
	Asian or Pacific Islander	0.48 (0.24, 0.71)***	0.01 (-0.27, 0.28)	-0.09 (-0.27, 0.09)	-0.05(-0.24, 0.15)	0.37 (0.10, 0.64)**
	Other or multiracial	0.31 (0.08, 0.55)**	-0.02 (-0.29, 0.25)	-0.01 (-0.19, 0.16)	-0.07(-0.26, 0.12)	0.13 (-0.14, 0.40)
Residency	In state	*Referent*	*Referent*	*Referent*	*Referent*	*Referent*
	Out of state	0.19 (0.02, 0.36)*	-0.03 (-0.23, 0.17)	0.01 (-0.12, 0.13)	-0.04 (-0.18, 0.10)	0.19 (-0.01, 0.38)
	International	0.16 (-0.15, 0.47)	0.18 (-0.17, 0.54)	-0.06 (-0.30, 0.17)	-0.06 (-0.31, 0.20)	0.27 (-0.09, 0.62)
College	Engineering	*Referent*	*Referent*	*Referent*	*Referent*	*Referent*
	Liberal Arts	-0.02 (-0.32, 0.28)	-0.16 (-0.51, 0.18)	0.06 (-0.16, 0.29)	-0.18 (-0.43, 0.07)	-0.10 (-0.44, 0.25)
	Health and Human Sciences	-0.07 (-0.32, 0.19)	0.03 (-0.27, 0.32)	0.09 (-0.10, 0.28)	0.13 (-0.08, 0.34)	-0.04 (-0.33, 0.25)
	Polytechnic Institute	-0.04 (-0.34, 0.25)	0.01 (-0.34, 0.35)	0.11 (-0.12, 0.34)	0.11 (-0.14, 0.36)	0.20 (-0.15, 0.54)
	Science	0.03 (-0.25, 0.30)	-0.21 (-0.53, 0.11)	-0.02 (-0.23, 0.19)	-0.06 (-0.29, .017)	0.01 (-0.31, 0.33)
	Other/more than one college	-0.24(-0.46, -0.03)*	-0.00(-0.25, 0.24)	-0.03 (-0.19, 0.13)	-0.01 (-0.18, 0.17)	-0.10 (-0.35, 0.14)
Class	Freshman	*Referent*	*Referent*	*Referent*	*Referent*	*Referent*
	Sophomore	0.21 (-0.03, 0.45)	0.02 (-0.25, 0.29)	0.16 (-0.02, 0.34)	0.04 (-0.15, 0.24)	0.11 (-0.16, 0.38)
	Junior	0.33 (0.08, 0.58)*	-0.16 (-0.44, 0.13)	0.14 (-0.05, 0.33)	-0.07 (-0.27, 0.14)	0.10 (-0.19, 0.39)
	Senior	0.30 (0.05, 0.55)*	-0.08 (-0.37, 0.21)	0.11 (-0.07, 0.30)	0.06 (-0.15, 0.26)	-0.07 (-0.36, 0.22)
	Graduate student	0.58 (0.36, 0.81)***	-0.21 (-0.47, 0.05)	0.25 (0.08, 0.42)**	-0.03 (-0.21, 0.16)	0.26 (-0.001, 0.52)
Know someone with COVID-19	No	*Referent*	*Referent*	*Referent*	*Referent*	*Referent*
	Family	-0.03 (-0.38, 0.32)	-0.37 (-0.78, 0.04)	-0.12 (-0.38, 0.15)	-0.36 (-0.65, -0.07)*	0.22 (-0.18, 0.63)
	Friend	-0.19 (-0.43, 0.04)	-0.44 (-0.70, -0.17)**	-0.10 (-0.27, 0.08)	-0.31 (-0.50, -0.12)**	-0.19 (-0.45, 0.08)
	Self	-0.74 (-1.38, -0.10)*	-0.44 (-1.17, 0.30)	-0.71 (-1.19, -0.22)**	-0.42 (-0.95, 0.11)	-1.00 (-1.74, -0.26)**
	Someone else	0.05 (-0.20, 0.29)	-0.29 (-0.57, -0.01)*	-0.06 (-0.25, 0.12)	-0.12 (-0.32, 0.08)	-0.11 (-0.39, 0.17)
	Multiple categories	-0.11 (-0.33, 0.11)	-0.43 (-0.69, -0.18)**	-0.18 (-0.35, -0.01)*	-0.34 (-0.52, -0.16)***	-0.21 (-0.46, 0.05)

^*^ *p* < 0.05; ** *p* < 0.01; *** *p* < 0.001. *N* = 641 for protocol effectiveness (self), *N* = 641 for protocol effectiveness (others), *N* = 643 for protocol adherence (self), *N* = 640 for protocol adherence (others), *N* = 641 for consequences of protocol violation. Values are β (95% confidence interval) from adjusted linear regression models. Models include all variables shown in the table as main effect covariates. A higher value indicates an opinion consistent with a higher level of prevention

**Table 3:** Adjusted difference in knowledge of violating guidelines or self-reported level of concern about COVID-19 associated with selected characteristics

Category	Variable	Knowledge of violating guidelines	Concern about COVID-19
Gender	Male	*Referent*	*Referent*
	Female	0.09 (-0.15, 0.32)	0.46 (0.18, 0.64)***
Race	White	*Referent*	*Referent*
	Asian or Pacific Islander	0.07 (-0.27, 0.41)	0.43 (0.17, 0.69)**
	Other or multiracial	0.17 (-0.17, 0.51)	0.24 (-0.02, 0.49)
Residency	In state	*Referent*	*Referent*
	Out of state	-0.01 (-0.25, 0.24)	0.22 (0.03, 0.41)*
	International	0.41 (-0.04, 0.86)	0.20 (-0.14, 0.53)
College	Engineering	*Referent*	*Referent*
	Liberal Arts	-0.16 (-0.59, 0.28)	0.24 (-0.09, 0.56)
	Health and Human Sciences	0.31 (-0.06, 0.68)	0.01 (-0.27, 0.29)
	Polytechnic Institute	-0.08 (-0.51, 0.36)	-0.08 (-0.41, 0.24)
	Science	0.15 (-0.25, 0.55)	0.16 (-0.13, 0.46)
	Other/more than one college	0.34 (0.03, 0.65)*	-0.08 (-0.31, 0.15)
Class	Freshman	*Referent*	*Referent*
	Sophomore	0.08 (-0.26, 0.43)	0.11 (-0.15, 0.37)
	Junior	0.16 (-0.20, 0.53)	0.37 (0.10, 0.64)**
	Senior	0.40 (0.03, 0.76)*	0.35 (0.07, 0.62)*
	Graduate student	-0.14 (-0.47, 0.18)	0.58 (0.33, 0.82)***
Know someone with COVID-19	No	*Referent*	*Referent*
	Family	0.32 (-0.19, 0.83)	0.20 (-0.18, 0.59)
	Friend	0.39 (0.06, 0.73)*	0.05 (-0.20, 0.30)
	Self	-0.72 (-1.64, 0.21)	-0.66 (-1.36, 0.03)
	Someone else	0.34 (-0.01, 0.69)	0.11 (-0.15, 0.37)
	Others/multiple	0.65 (0.33, 0.97)***	0.12 (-0.12, 0.36)

*N* = 643 for knowledge of violating guidelines; *N* = 643 for concern about covid-19. Values are β (95% confidence interval) from adjusted linear regression models. Models include all variables shown in the table. Bold type indicates *p* < 0.05 for result compared to referent. A higher value indicates an opinion consistent with a higher level of prevention

Scores for protocol adherence (self) were significantly higher among graduate students versus freshman in multiple linear regression analysis (Table 2). However, participants who reported having a positive COVID-19 test and multiple categories of known COVID-19 individuals (versus reporting none) had significantly lower scores in protocol adherence (self). For protocol adherence (others), we found significantly lower score in participants who knew family members, friends or multiple categories of known people who tested positive for COVID-19 versus reporting none. Scores for consequences of protocol violation were significantly higher among females (versus males) and Asian/Pacific Islanders (versus white participants); however, they were significantly lower among participants who tested positive for COVID-19 themselves (versus reporting none).

In multiple linear regression analyses, scores were significantly higher for knowledge of violating guidelines for participants in other or multiple colleges (versus engineering), seniors (versus freshman), as well as those reporting that a friend or others/ multiple categories tested positive for COVID-19 (versus those who reported none) (Table 3). Scores for concern about COVID-19 were significantly higher in females (versus males), Asian/ Pacific Islander (versus White), out-of-state (versus in state) as well as juniors, seniors, or graduate students (versus freshman).

## Discussion

In this study, we found that characteristics most likely to be correlated with higher belief in or adherence to preventive measures were female, Asian/Pacific Islanders or graduate students. Meanwhile, respondents who reported knowing someone who had COVID-19 tended to report lower belief in or adherence to these preventive measures.

Female participants showed higher belief in or adherence to protective measures compared to their male counterparts. This is consistent with several previous surveys also conducted during the COVID-19 pandemic which report that females followed protective guidelines [18–21] and had a higher perception of risk compared to males [20]; these trends were also seen among studies conducted in university settings[29, 33]. Consistent with these observations, males have also been reported to practice riskier behaviors than females [36].

Higher level undergraduates and graduate participants showed higher belief and adherence with the preventive measures than the younger students; similar to results from results from a multi-university study from Albaqawi and colleagues [29]. This could reflect differences in age or in educational levels. One study conducted in Germany found that men with more education were more worried about COVID-19 than the men with less education [37]. Level of education has also been found to be a significant factor impacting the knowledge and perceptions of the Nipah Virus in Bangladesh [38]. Several other studies have found correlations of age with taking preventive measures. For example, Luo et al. report that older generation are more likely to take preventive measures than younger generation [39].

Several outcomes were significantly higher among self-reported Asian/Pacific Islanders. This is consistent with prior literature noting that non-white participants were more likely to wear masks [15, 16]. However, there were insufficient data to explore these association with other self-reported racial/ethnic groups in our study.

There were several limitations to this study. First, data were collected solely from Purdue students, so results may not be generalizable to other settings. It is also possible that response bias could have influenced our results. However, many of our key findings were similar to those reported by studies conducted in the general population, which suggests that there is at least some degree of generalizability of our results. Additionally, this analysis is based on self-reported data, but we are not aware of any potential bias that could influence these data. It is possible that there could be some difference in response related to calendar time, as the survey was available over a few months and our knowledge of COVID-19 was rapidly changing throughout this period. However, campus COVID-19 prevention policies on campus did not change over the course of the data collection period.

There are also several strengths to this study. There have been few studies of COVID-19 prevention within institutions of higher education to date. Increased knowledge for this specific setting is important as a university community may be highly vulnerable to disease outbreaks as they typically have a large amount of social interaction and travel. Several prior studies have documented disease outbreaks focused within university communities [40–48]. Additionally, our results provide more information regarding which characteristics are strongly correlated with student belief and adherence to prevention protocols, which may help administrators in the design and application of any future policies related to infectious disease prevention.

At the time of writing, incidence of COVID-19 has decreased to a point where many universities and institutions, including Purdue, have currently relaxed their preventive measures. However, the emergence of COVID-19 variants or other emerging infectious diseases is a possibility [49, 50], and these could trigger reinstatement of similar protocols. Results from this study and similar studies are promising in that they suggest that the policies used in the COVID-19 pandemic were largely effective. However, improvements could be made in education and/or risk communication to men, younger students, and students with a close contact that was ill.

## Conclusions

Results from our online survey of Purdue University students are largely consistent with prior literature related to perceptions and knowledge of COVID-19 or other infectious disease prevention measures. Females, Asians/Pacific Islanders, and graduate students reported higher belief and adherence in the COVID-19 prevention measures..

## Supplementary Information

**Additional file 1.**

## Data Availability

The datasets used and/or analyzed during the current study available from the corresponding author on reasonable request.

## References

[1] Zhu N, Zhang D, Wang W, Li X, Yang B, Song J (2020). A Novel Coronavirus from Patients with Pneumonia in China, 2019. N Engl J Med.

[2] Sohrabi C, Alsafi Z, O’Neill N, Khan M, Kerwan A, Al-Jabir A (2020). World Health Organization declares global emergency: A review of the 2019 novel coronavirus (COVID-19). Int J Surg.

[3] Wiersinga WJ, Rhodes A, Cheng AC, Peacock SJ, Prescott HC (2020). Pathophysiology, Transmission, Diagnosis, and Treatment of Coronavirus Disease 2019 (COVID-19) A Review. Jama-J Am Med Assoc.

[4] Chu DK, Akl EA, Duda S, Solo K, Yaacoub S, Schunemann HJ (2020). Physical distancing, face masks, and eye protection to prevent person-to-person transmission of SARS-CoV-2 and COVID-19: a systematic review and meta-analysis. Lancet.

[5] Viner RM, Russell SJ, Croker H, Packer J, Ward J, Stansfield C (2020). School closure and management practices during coronavirus outbreaks including COVID-19: a rapid systematic review. Lancet Child Adolesc Health.

[6] Nussbaumer-Streit B, Mayr V, Dobrescu AI, Chapman A, Persad E, Klerings I, et al. Quarantine alone or in combination with other public health measures to control COVID-19: a rapid review. Cochrane Database Syst Rev. 2020;9:CD013574. https://pubmed.ncbi.nlm.nih.gov/33959956/. 10.1002/14651858.CD013574.pub2 PMC8133397 33959956

[7] Budd J, Miller BS, Manning EM, Lampos V, Zhuang M, Edelstein M (2020). Digital technologies in the public-health response to COVID-19. Nat Med.

[8] Pereira FMV, Lam SC, Chan JHM, Malaguti-Toffano SE, Gir E (2015). Difference in compliance with Standard Precautions by nursing staff in Brazil versus Hong Kong. Am J Infect Control.

[9] Evanoff B, Kim L, Mutha S, Jeffe D, Haase C, Andereck D (1999). Compliance with universal precautions among emergency department personnel caring for trauma patients. Ann Emerg Med.

[10] Ganczak M, Szych Z (2007). Surgical nurses and compliance with personal protective equipment. J Hosp Infect.

[11] Nichol K, Bigelow P, O’Brien-Pallas L, McGeer A, Manno M, Holness DL (2008). The individual, environmental, and organizational factors that influence nurses’ use of facial protection to prevent occupational transmission of communicable respiratory illness in acute care hospitals. Am J Infect Control.

[12] Brooks SK, Greenberg N, Wessely S, Rubin GJ (2021). Factors affecting healthcare workers’ compliance with social and behavioural infection control measures during emerging infectious disease outbreaks: rapid evidence review. BMJ Open.

[13] Lau JTF, Griffiths S, Choi K, Lin C (2010). Prevalence of preventive behaviors and associated factors during early phase of the H1N1 influenza epidemic. Am J Infect Control.

[14] Akan H, Gurol Y, Izbirak G, Ozdatlı S, Yilmaz G, Vitrinel A (2010). Knowledge and attitudes of university students toward pandemic influenza: a cross-sectional study from Turkey. BMC Public Health.

[15] Rubin GJ, Amlôt R, Page L, Wessely S (2009). Public perceptions, anxiety, and behaviour change in relation to the swine flu outbreak: cross sectional telephone survey. BMJ.

[16] Seale H, Heywood AE, McLaws M-L, Ward KF, Lowbridge CP, Van D (2010). Why do I need it? I am not at risk! Public perceptions towards the pandemic (H1N1) 2009 vaccine. BMC Infect Dis.

[17] Bish A, Michie S (2010). Demographic and attitudinal determinants of protective behaviours during a pandemic: A review. Br J Health Psychol.

[18] Abdelrahman M (2020). Personality Traits, Risk Perception, and Protective Behaviors of Arab Residents of Qatar During the COVID-19 Pandemic. Int J Ment Health Addict.

[19] Ferdous Mostz, Islam MdS, Sikder MdT, Mosaddek ASMd, Zegarra-Valdivia JA, Gozal D (2020). Knowledge, attitude, and practice regarding COVID-19 outbreak in Bangladesh: An online-based cross-sectional study. Plos One.

[20] Rana IA, Bhatti SS, Aslam AB, Jamshed A, Ahmad J, Shah AA (2021). COVID-19 risk perception and coping mechanisms: Does gender make a difference?. Int J Disaster Risk Reduct.

[21] Zhong B-L, Luo W, Li H-M, Zhang Q-Q, Liu X-G, Li W-T (2020). Knowledge, attitudes, and practices towards COVID-19 among Chinese residents during the rapid rise period of the COVID-19 outbreak: a quick online cross-sectional survey. Int J Biol Sci.

[22] Perić M, Wise N, Heydari R, Keshtidar M, Mekinc J (2021). Getting back to the event: COVID-19, attendance andperceived importance of protective measures <b/>. Kinesiology.

[23] Mueller AS, Diefendorf S, Abrutyn S, Beardall KA, Millar K, O’Reilly L (2021). Youth Mask-Wearing and Social-Distancing Behavior at In-Person High School Graduations During the COVID-19 Pandemic. J Adolesc Health Off Publ Soc Adolesc Med.

[24] Hast M, Swanson M, Scott C, Oraka E, Espinosa C, Burnett E (2022). Prevalence of risk behaviors and correlates of SARS-CoV-2 positivity among in-school contacts of confirmed cases in a Georgia school district in the pre-vaccine era, December 2020-January 2021. BMC Public Health.

[25] Barrios LC, Riggs MA, Green RF, Czarnik M, Nett RJ, Staples JE (2021). Observed Face Mask Use at Six Universities - United States, September-November 2020. MMWR Morb Mortal Wkly Rep.

[26] Burnell K, Robbins M, Kulali S, Wells EM (2022). Prevalence and predictors of mask use on a large US university campus during the COVID-19 pandemic: A brief report. Am J Infect Control.

[27] Hatami H, Abbasi-Kangevari M, Malekpour M-R, Kolahi A-A (2021). Knowledge, Attitudes, and Safety Practices About COVID-19 Among High School Students in Iran During the First Wave of the Pandemic. Front Public Health.

[28] Naseef HA, Al-Shami NA, Abu Hadba LS, Humos LA, Shaheen RN, Mitwasi TT, et al. Knowledge, attitudes, and practices about coronavirus disease (COVID-19) among Birzeit University students: a cross-sectional study. J Public Health-Heidelb. 10.1007/s10389-021-01665-0. 10.1007/s10389-021-01665-0 PMC8574150 34777949

[29] Albaqawi HM, Alquwez N, Balay-odao E, Bajet JB, Alabdulaziz H, Alsolami F (2020). Nursing Students’ Perceptions, Knowledge, and Preventive Behaviors Toward COVID-19: A Multi-University Study. Front Public Health.

[30] Fatima M, Habib A, Khan S, Butt MH, Mallhi TH, Khan YH, et al. Knowledge, Attitude, Practice, Behavior and Risk Perception of COVID-19 Pandemic among Medical and non-Medical University Students. Disaster Med Public Health Prep. 2022:1–4. https://pubmed.ncbi.nlm.nih.gov/35000664/. 10.1017/dmp.2022.1 PMC8886059 35000664

[31] Jia Y, Ma S, Bai L, Xiao Q, Wu Y, Gao Y (2021). Health Literacy and Disparities in Knowledge, Attitude and Practice Regarding COVID-19 Among College Students During the COVID-19 Outbreak in China: A Cross-Sectional Study. Risk Manag Healthc Policy.

[32] Middleton N, Tsioutis C, Kolokotroni O, Heraclides A, Theodosis-Nobelos P, Mamais I (2021). Gaps in Knowledge About SARS-CoV-2 & COVID-19 Among University Students Are Associated With Negative Attitudes Toward People With COVID-19: A Cross-Sectional Study in Cyprus. Front Public Health.

[33] Rodriguez-Besteiro S, Tornero-Aguilera JF, Fernandez-Lucas J, Clemente-Suarez VJ (2021). Gender Differences in the COVID-19 Pandemic Risk Perception, Psychology, and Behaviors of Spanish University Students. Int J Environ Res Public Health.

[34] Emrani J, Hefner EN. Socio-demographic Heterogeneity in Prevalence of SARS-COV-2 Infection and Death Rate: Relevance to Black College Student Knowledge of COVID-19 and SARS-COV-2. J Racial Ethn Health Disparities. 10.1007/s40615-021-01193-3. 10.1007/s40615-021-01193-3 PMC8815385 35119679

[35] Cherney E. Universities are suspending students who party without masks and flout social distancing: ‘If you don’t abide by the rules, there’s no place for you here.’ chicagotribune.com. https://www.chicagotribune.com/coronavirus/ct-covid-19-illinois-college-students-suspended-20200902-qx64j6opvvge5phwitvl6dz2qu-story.html. Accessed 2 Aug 2021.

[36] Bates BR, Moncayo AL, Costales JA, Herrera-Cespedes CA, Grijalva MJ (2020). Knowledge, Attitudes, and Practices Towards COVID-19 Among Ecuadorians During the Outbreak: An Online Cross-Sectional Survey. J Community Health.

[37] Rattay P, Michalski N, Domanska OM, Kaltwasser A, De Bock F, Wieler LH (2021). Differences in risk perception, knowledge and protective behaviour regarding COVID-19 by education level among women and men in Germany. Results from the COVID-19 Snapshot Monitoring (COSMO) study. Plos One.

[38] Hassan MM, Kalam MdA, Alam M, Shano S, Faruq AA, Hossain MdS (2020). Understanding the Community Perceptions and Knowledge of Bats and Transmission of Nipah Virus in Bangladesh. Animals.

[39] Luo Y, Cheng Y, Sui M (2021). The Moderating Effects of Perceived Severity on the Generational Gap in Preventive Behaviors during the COVID-19 Pandemic in the U.S.. Int J Environ Res Public Health.

[40] Choe YJ, Park Y-J, Kim JW, Eom HE, Park O, Oh M (2017). An Outbreak of Measles in a University in Korea, 2014. J Korean Med Sci.

[41] Greenland K, Whelan J, Fanoy E, Borgert M, Hulshof K, Yap K-B (2012). Mumps outbreak among vaccinated university students associated with a large party, the Netherlands, 2010. Vaccine.

[42] Iuliano AD, Reed C, Guh A, Desai M, Dee DL, Kutty P (2009). Notes from the Field: Outbreak of 2009 Pandemic Influenza A (H1N1) Virus at a Large Public University in Delaware, April–May 2009. Clin Infect Dis.

[43] Kirolos A, Waugh C, Templeton K, McCormick D, Othieno R, Willocks LJ (2018). Imported case of measles in a university setting leading to an outbreak of measles in Edinburgh, Scotland from September to December 2016. Epidemiol Infect.

[44] Layde PM, Engelberg AL, Dobbs HI, Curtis AC, Craven RB, Graitcer PL (1980). Outbreak of Influenza A/USSR/77 at Marquette University. J Infect Dis.

[45] Patel LN, Arciuolo RJ, Fu J, Giancotti FR, Zucker JR, Rakeman JL, et al. Mumps Outbreak Among a Highly Vaccinated University Community—New York City, January–April 2014. Clin Infect Dis. 2017;4:408–12. https://pubmed.ncbi.nlm.nih.gov/27927872/. 10.1093/cid/ciw762 27927872

[46] Rota JS, Turner JC, Yost-Daljev MK, Freeman M, Toney DM, Meisel E (2009). Investigation of a mumps outbreak among university students with two measles-mumps-rubella (MMR) vaccinations, Virginia, September-December 2006. J Med Virol.

[47] Shah M, Quinlisk P, Weigel A, Riley J, James L, Patterson J (2018). Mumps Outbreak in a Highly Vaccinated University-Affiliated Setting Before and After a Measles-Mumps-Rubella Vaccination Campaign—Iowa, July 2015–May 2016. Clin Infect Dis.

[48] Waller JL, Diaz MH, Petrone BL, Benitez AJ, Wolff BJ, Edison L (2014). Detection and Characterization of Mycoplasma pneumoniae during an Outbreak of Respiratory Illness at a University. J Clin Microbiol.

[49] Telenti A, Arvin A, Corey L, Corti D, Diamond MS, García-Sastre A (2021). After the pandemic: perspectives on the future trajectory of COVID-19. Nature.

[50] Clark H, Sirleaf EJ (2021). Ending this pandemic and securing the future. BMJ.

