# Supplementary material for: Online survey of university students’ perception, awareness and adherence to COVID-19 prevention measures
Source: BMC Public Health. 2022 May 13;22:964. doi: 10.1186/s12889-022-13356-w (PMC9103602; doi:10.1186/s12889-022-13356-w)
Supplement: Supplementary file 1 — Additional file 1. [file 12889_2022_13356_MOESM1_ESM.pdf]

SUPPLEMENTAL INFORMATION

Online Survey of University Students' Perception, Awareness and Adherence to COVID-19 Prevention Measures

Salma Akhter,[1*] Meredith Robbins,[1*] Perry Curtis,[1] Belle Hinshaw,[1] Ellen M. Wells[1,2,#]

1. School of Health Sciences, Purdue University, West Lafayette, Indiana, USA
2. Department of Public Health, Purdue University, West Lafayette, Indiana, USA

[*] These authors made equal contributions to this work.

SUPPLEMENT

COVID-19 Prevention Measures Survey

What is your gender?
- Male
- Female
- Other, specify: _____________

Which of the following best describes you?
- Asian or Pacific Islander
- Black or African American
- Hispanic or Latino
- Native American or Alaskan
- White or Caucasian
- Multiracial or Biracial
- Other, specify: ___________

Which option best describes your permanent residence?
- In-state
- Out-of-state
- International

Which class are you in?
- Freshman
- Sophomore
- Junior
- Senior
- 5th year senior
- Graduate student

Which college are you in? *(select all that apply)*
- College of Agriculture
- College of Education
- College of Engineering
- Exploratory Studies
- College of Health and Human Sciences
- College of Liberal Arts
- Krannert School of Management
- College of Pharmacy
- Purdue Polytechnic Institute
- College of Science
- College of Veterinary Medicine

Do you know anyone who has tested positive for COVID-19? *(select all that apply)*
- No
- Yes, I have tested positive
- Yes, a family member tested positive
- Yes, a friend tested positive
- Yes, someone else tested positive

How concerned are you about COVID-19?
- Not at all concerned
- Somewhat concerned
- Moderately concerned
- Very concerned
- Extremely concerned

Please indicate your level of agreement with the following statements (1-disagree; 2-somewhat disagree; 3-neither agree nor disagree; 4-somewhat agree; 5-agree):

**Statement**	**1**	**2**	**3**	**4**	**5**
I am comfortable not socially distancing if I have a mask on.					
I am comfortable not wearing a mask if I am social distancing.					
I don't feel like I need to wear a mask if I am using a face shield.					
I believe that social distancing and/or wearing a mask are effective in reducing my risk from COVID-19.					
I feel that people around me understand the importance of social distancing.					
I feel that people around me understand the importance of wearing a mask.					
I usually follow the Protect Purdue guidelines.					
My friends usually follow the Protect Purdue guidelines.					
Other students usually follow the Protect Purdue guidelines.					
I am aware of students who are hosting social events that violate the Protect Purdue Guidelines.					
I would feel comfortable anonymously reporting someone for violating the Protect Purdue Guidelines.					
I would feel comfortable anonymously reporting a friend for violating the Protect Purdue Guidelines.					
I agree that suspension and other forms of disciplinary action are appropriate for those who violate the Protect Purdue guidelines.					

Do you have any additional thoughts or concerns about wearing masks or social distancing that you would like to share? ____________________

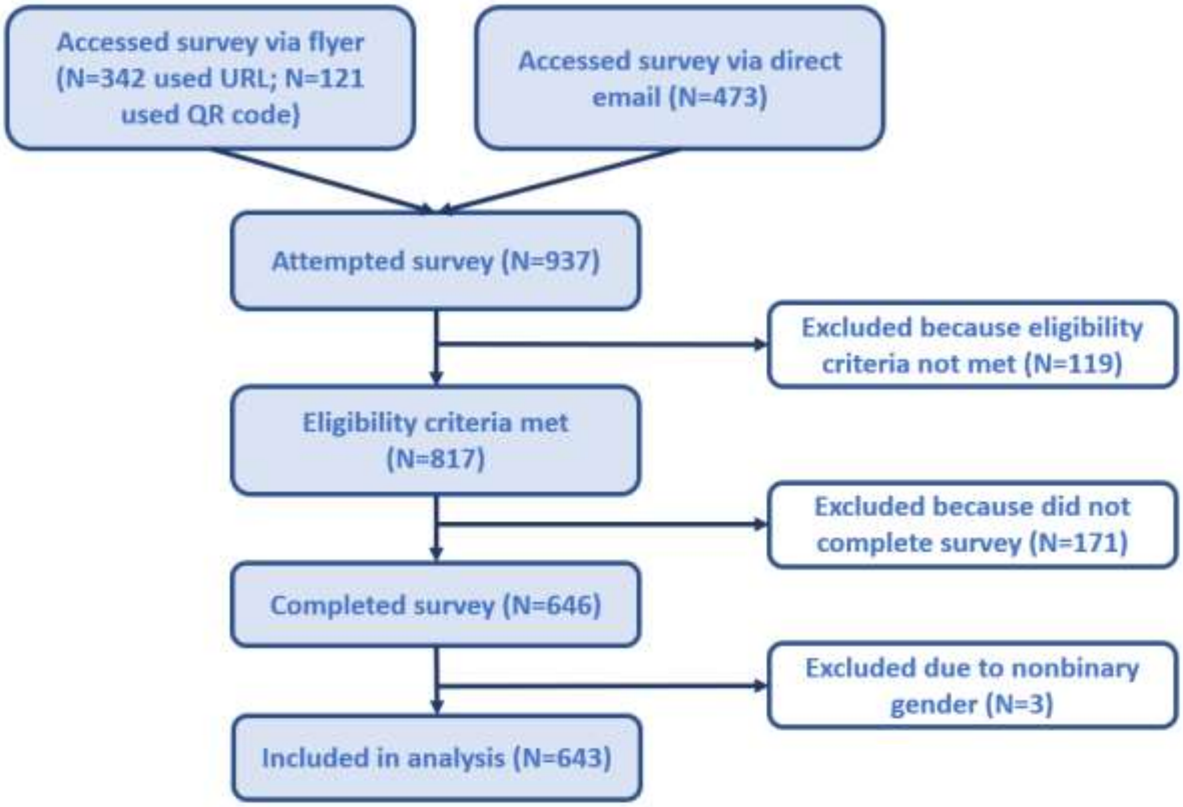

Figure S1: Flow chart of participants.

Table S1. Categorization of individual survey questions about the COVID-19 protocol

Category	Statements	Mean Score (SD)
Protocol effectiveness (self)	I am comfortable not socially distancing if have a mask on.	2.63 (1.34) [a]
	I am comfortable not wearing a mask if I am social distancing.	3.03 (1.48) [a]
	I don't feel like I need to wear a mask if I am using a face shield.	3.73 (1.37) [a]
	I believe that social distancing and/or wearing a mask are effective in reducing my risk from COVID-19.	4.43 (0.97)
Protocol effectiveness (others)	I feel that people around me understand the importance of social distancing.	3.32 (1.15)
	I feel that people around me understand the importance of wearing a mask.	3.58 (1.16)
Protocol adherence (self)	I usually follow the Protect Purdue guidelines.	4.60 (0.71)
Protocol adherence (others)	My friends usually follow the Protect Purdue guidelines.	4.28 (0.82)
	Other students usually follow the Protect Purdue guidelines.	3.54 (1.06)
Consequences of protocol violation	I would feel comfortable anonymously reporting someone for violating the Protect Purdue Guidelines.	3.11 (1.32)
	I would feel comfortable anonymously reporting a friend for violating the Protect Purdue Guidelines.	2.66 (1.32)
	I agree that suspension and other forms of disciplinary action are appropriate for those who violate the Protect Purdue guidelines.	3.60 (1.34)

SD = standard deviation. Respondents indicated their agreement with each statements on a scale of 1 (strongly disagree) to 5 (strongly agree).

a. Scores for these questions were recategorized to 1 (strongly agree) to 5 (strongly disagree) so that the direction of all responses would consistently have a higher score represent a response consistent with increased support for the protective measures.

Table S2. Unadjusted relationship of demographic characteristics with student opinions regarding prevention guidelines

Category	Variable	Protocol effectiveness (self)	Protocol effectiveness (others)	Protocol adherence (self)	Protocol adherence (others)	Consequences of protocol violation
Gender	Male	Referent	Referent	Referent	Referent	Referent
	Female	0.19 (0.03, 0.35)*	-0.28 (-0.45, -0.11)**	0.09 (-0.02, 0.21)	-0.10 (-0.23,0.02)	0.14(-0.04, 0.32)
Race	White	Referent	Referent	Referent	Referent	Referent
	Asian or Pacific Islander	0.67 (0.46, 0.88)***	0.21 (-0.03, 0.44)	-0.04 (-0.19, 0.12)	0.05 (-0.12, 0.22)	0.56(0.33, 0.80)***
	Other	0.38 (0.15, 0.62)**	0.01 (-0.25, 0.27)	-0.00 (-0.17, 0.17)	-0.08 (-0.27, 0.11)	0.23(-0.03, 0.50)
Residency	In state	Referent	Referent	Referent	Referent	Referent
	Out of state	0.27 (0.10, 0.44)**	0.03 (-0.16, 0.22)	-0.01 (-0.13, 0.12)	-0.02 (-0.15, 0.12)	0.24(0.05, 0.43)*
	International	0.65 (0.38, 0.92)***	0.29 (-0.01, 0.59)	-0.02 (-0.22, 0.17)	-0.00 (-0.22, 0.21)	0.62(0.32, 0.93)***
College	Engineering	Referent	Referent	Referent	Referent	Referent
	Liberal Arts	-0.05 (-0.36, -0.27)	-0.30 (-0.64, 0.04)	0.09 (-0.13, 0.31)	-0.24 (-0.48, 0.00)	-0.14(-0.48, 0.21)
	Health and Human Sciences	-0.07 (-0.32, 0.18)	-0.18 (-0.45, 0.09)	0.10 (-0.07, 0.28)	0.03 (-0.17, 0.22)	-0.08(-0.35, 0.20)
	Polytechnic Institute	-0.28 (-0.59, 0.03)	0.02 (-0.32, 0.36)	0.05 (-0.17, 0.27)	0.10 (-0.14, 0.35)	0.04(-0.31, 0.39)
	Science	-0.05 (-0.33, 0.24)	-0.22 (-0.54, 0.09)	-0.04 (-0.24, 0.17)	-0.06 (-0.29, 0.16)	-0.05(-0.37, 0.27)
	Other / more than one college	-0.28 (-0.50, -0.07)	-0.10 (-0.33, 0.13)	-0.03 (-0.18, 0.12)	-0.06 (-0.23, 0.10)	-0.13(-0.36, 0.11)
Class	Freshman	Referent	Referent	Referent	Referent	Referent
	Sophomore	0.26 (0.01, 0.50)	0.04 (-0.23, 0.32)	0.14 (-0.04, 0.31)	0.04 (-0.16, 0.23)	0.11(-0.16, 0.38)
	Junior	0.37 (0.11, 0.63)**	-0.15 (-0.43, 0.14)	0.15 (-0.04, 0.33)	-0.07 (-0.27, 0.13)	0.11(-0.18, 0.40)
	Senior	0.32 (0.07, 0.58)*	-0.05 (-0.34, 0.23)	0.12 (-0.06, 0.31)	0.08 (-0.13, 0.28)	-0.06 (-0.35, 0.20)
	Graduate student	0.69 (0.47, 0.91)***	-0.10 (-0.35, 0.15)	0.22 (0.06, 0.38)**	-0.03 (-0.20, 0.15)	0.36 (0.11, 0.61)**
Know someone with COVID-19	No	Referent	Referent	Referent	Referent	Referent
	Family	-0.09 (-0.45, 0.28)	-0.43 (-0.83, -0.03)*	-0.05 (-0.31, 0.21)	-0.34 (-0.63, -0.06)*	0.16 (-0.24, 0.56)*
	Friend	-0.33 (-0.56, -0.10)**	-0.48 (-0.73, -0.22)***	-0.05 (-0.21, 0.12)	-0.28 (-0.47, -0.10)**	-0.30 (-0.56, -0.04)**
	Self	-0.98 (-1.65, -0.31)**	-0.46 (-1.19, 0.27)	-0.67 (-1.16, -1.19)**	-0.40 (-0.92, 0.13)	-1.12(-1.87, -0.38)
	Someone else	-0.04 (-0.29, 0.21)	-0.32 (-0.59, -0.04)*	-0.03 (-0.21, 0.14)	-0.11 (-0.30, 0.08)	-0.17(-0.44, 0.11)
	Others/multiple	-0.29 (-0.51, -0.07)*	-0.47 (-0.71, -0.24)***	-0.13 (-0.28, 0.03)	-0.31 (-0.48, -0.15)***	-0.36(-0.60, -0.12)**

* $p < 0.05$; ** $p < 0.01$; *** $p < 0.001$. N=641 for protocol effectiveness (self); N= 641 for protocol effectiveness (others); N=643 for protocol adherence (self); N= 640 for protocol adherence (others); N=641 for consequences of protocol violation. Values are β (95% confidence interval) from unadjusted linear regression models. A higher value indicates an opinion consistent with a higher level of prevention.

Table S3. Unadjusted relationship of demographic characteristics with knowledge of violating guidelines or self-reported level of concern about COVID-19

Category	Variable	Knowledge of violating guidelines	Concern about COVID-19
Gender	Male	Referent	Referent
	Female	-0.22 (-0.004, 0.44)	0.41 (0.24, 0.58)***
Race	White	Referent	Referent
	Asian or Pacific Islander	-0.04 (-0.34, 0.27)	0.51 (0.28, 0.74)***
	Other	0.18 (-0.16, 0.51)	0.32 (0.06, 0.57)*
Residency	In state	Referent	Referent
	Out of state	-0.06 (-0.31, 0.18)	0.23 (0.04, 0.41)*
	International	0.07 (-0.31, 0.46)	0.52 (0.22, 0.82)**
College	Engineering	Referent	Referent
	Liberal Arts	-0.13 (-0.56, 0.30)	0.28 (-0.05, 0.61)
	Health and Human Sciences	0.47 (0.13, 0.82)**	0.15 (-0.12, 0.41)
	Polytechnic Institute	-0.10 (-0.53, 0.34)	-0.30 (-0.63, 0.04)
	Science	0.16 (-0.24, 0.56)	0.10 (-0.20, 0.42)
	Other/more than one college	0.39 (0.09, 0.68)*	-0.05 (-0.28, 0.18)
Class	Freshman	Referent	Referent
	Sophomore	0.10 (-0.25, 0.44)	0.13 (-0.13, 0.39)
	Junior	0.15 (-0.21, 0.52)	0.40 (0.13, 0.68)**
	Senior	0.45 (0.09, 0.82)*	0.35 (0.08, 0.63)*
	Graduate student	-0.09 (-0.41, 0.22)	0.65 (0.41, 0.89)***
Know someone with COVID-19	No	Referent	Referent
	Family	0.34 (-0.16, 0.85)	0.18 (-0.21, 0.58)
	Friend	0.43 (0.11, 0.75)**	-0.04 (-0.29, 0.22)
	Self	-0.82 (-1.75, 0.11)	-0.87 (-1.60, -0.13)*
	Someone else	0.30 (-0.04, 0.65)	0.05 (-0.22, 0.32)
	Others/multiple	0.67 (0.37, 0.97)***	-0.01 (-0.25, 0.23)

* $p < 0.05$; ** $p < 0.01$; *** $p < 0.001$. N= 643 for knowledge of violating guidelines; N= 643 for concern about covid-19. Values are β (95% confidence interval) from unadjusted linear regression models. A higher value indicates an opinion consistent with a higher level of prevention.
